# Regulation of Hxt3 and Hxt7 Turnover Converges on the Vid30 Complex and Requires Inactivation of the Ras/cAMP/PKA Pathway in *Saccharomyces cerevisiae*

**DOI:** 10.1371/journal.pone.0050458

**Published:** 2012-12-05

**Authors:** Chris Snowdon, George van der Merwe

**Affiliations:** Department of Molecular and Cellular Biology, University of Guelph, Guelph, Ontario, Canada; Institute of Biology Valrose, France

## Abstract

Eukaryotic cells adjust their intracellular protein complement as a mechanism to adapt to changing environmental signals. In *Saccharomyces cerevisiae* the hexose transporters Hxt3 and Hxt7 are expressed and function on the plasma membrane in high and low glucose abundance, respectively. By contrast, Hxt3 is endocytosed and degraded in the vacuole when cells are starved of glucose and Hxt7 in response to rapamycin treatment or when nitrogen is limiting. Yeast uses several signaling pathways, including the TORC1 and Ras/cAMP/Protein Kinase A (PKA) pathways, to adapt to nutrient changes in the environment. The multi-protein Vid30 complex (Vid30c), an E3 ubiquitin ligase required for the degradation of FBPase, assists in this adaptation process in a mechanism that is poorly understood. Here we show the endocytosis and the subsequent degradation of both Hxt3 and Hxt7, in response to different nutrient signals, is dependent on components of the Vid30c. Additionally, we define the signaling events required for the turnover of Hxt3 and Hxt7 by showing that Hxt3 turnover requires Ras2 and PKA inactivation, whereas Hxt7 turnover requires TORC1 and Ras2 inactivation. Further investigation led us to identify Rim15, a kinase that is inhibited by both the TORC1 and Ras/cAMP/PKA pathways, as a key downstream effector in signaling both turnover events. Finally, we show that the turnover of both Hxt3 and Hxt7 is dependent on the essential E3 ubiquitin ligase, Rsp5, indicating that the role of the Vid30c might be indirect of Hxt ubiquitylation.

## Introduction

The Target Of Rapamycin (TOR) and Ras/cAMP/Protein Kinase A (PKA) signaling pathways enable *Saccharomyces cerevisiae* to respond to nutrient availability and stress [1]–[4]. The two TOR kinases, Tor1 and Tor2, are pivotal proteins in the TORC1 signaling cascade that has wide-ranging effects in the cell. Rich nutrient conditions activate TORC1 to promote cell cycle progression and protein synthesis, while preventing autophagy and regulating the expression of metabolic genes in response to nutrient availability, and inhibiting the expression of stress response genes. By contrast, TORC1 is inactivated by nutrient starvation or rapamycin treatment resulting in cell cycle arrest, a decrease in protein synthesis, the activation of autophagy, and the increased expression of stress response and nitrogen-regulated genes [1], [4]–[9]. Similarly, the Ras/cAMP/PKA pathway also antagonizes stress response and promotes cell proliferation in the absence of stress and in the presence of abundant glucose [3], [10]. Glucose limitation and cell stress inactivate this pathway leading to cell cycle arrest, the synthesis of complex carbohydrates, the activation of stress response genes, and the derepression of glucose repressed genes [3], [11], [12]. Interestingly, these two distinct pathways show a level of cross communication, as TOR signaling has been shown to converge on similar targets as the Ras/cAMP/PKA pathway [13], [14].

The activity of PKA is controlled by intracellular cAMP [15]. In the presence of glucose, the two redundant small G proteins Ras1 and Ras2 are activated via the guanine exchange factors Cdc25 and Sdc25 [16], [17]. Active Ras1/2 in turn activates the adenylyl cyclase, Cyr1, to produce cAMP [18]. The presence of cAMP activates PKA by releasing it from its inhibitory interaction with the regulatory subunit Bcy1 [15]. The activity of Ras1/2 is negatively modulated by the GTPase activating proteins Ira1 and Ira2 [19], [20], while the intracellular level of cAMP is controlled by the phosphodiesterases Pde1 and Pde2 [21], [22]. Active PKA prevents cell cycle arrest, post diauxic shift gene expression and glycogen accumulation by phosphorylating and inactivating Rim15, a kinase essential for the activation of these processes [14], [23]. Conversely, in the absence of glucose or in response to stress, the decrease in cAMP allows for Bcy1 to bind and inactivate PKA, resulting in the activation of Rim15 [13].

Hexose transporters are regulated at the transcriptional and post-translational levels to allow yeast to adapt to varying nutrient concentrations in the environment. If conditions become unfavorable for the expression of a specific transporter gene, the cell must repress its transcription and degrade the remaining transporter. This degradation occurs via endocytosis and proteolysis in the vacuole. For example, *HXT7* encodes a high affinity hexose transporter and its transcription is induced by low levels of glucose or a non-fermentable carbon source and Hxt7 localizes to the plasma membrane. However, in response to glucose abundance, nitrogen starvation or rapamycin treatment *HXT7* transcription is repressed and Hxt7 is degraded in the vacuole [24], [25]. By contrast, *HXT3* encodes a low affinity hexose transporter that is actively expressed in glucose abundance, but repressed [26] and the gene product degraded when only a non-fermentable carbon source like ethanol is supplied [27]. Despite much research into the turnover of hexose transporters, the signaling and regulatory mechanisms that govern this process are not fully understood.

The Vid/Gid proteins play an important role in the yeast’s adaptation to different nutrient conditions. These proteins assemble into a multi-component complex termed the Vid30 complex (Vid30c) [28] that functions as an E3 ubiquitin ligase [29], [30] able to facilitate the ubiquitin-dependent degradation of FBPase and Mdh2 following the transition from gluconeogenic to glycolytic growth conditions [31], [32]. Interestingly, at least three of these proteins, Vid30, Gid2 and Vid28, are needed for the turnover of Hxt7 upon nitrogen starvation or rapamycin treatment, and the growth of several *vid/gid* mutants are sensitive to the presence of rapamycin in the media [25]. Also, the transcription of the *VID*/*GID* genes increases in the presence of non-fermentable carbon sources [26]. The function(s) of the Vid30c therefore seems to correlate with the presence of poor carbon and nitrogen sources.

Here we further investigate the link between the Vid30c and the regulatory mechanisms that govern hexose transporter (Hxt) turnover. We expand the known function of the Vid30c in the nitrogen starvation and rapamycin-induced turnover of Hxt7 [25] to include the glucose starvation-induced degradation of Hxt3. Additionally, we show that signaling the condition-specific turnover of both these Hxts requires inactivation of the Ras/cAMP/PKA pathway thereby activating Rim15 to facilitate the turnover process. Finally, we demonstrate that Rsp5, an essential E3 ubiquitin ligase known to directly ubiquitylate nutrient transporters [33]–[35], is critical for the endocytosis and degradation of both Hxt3 and Hxt7.

**Figure 1:**
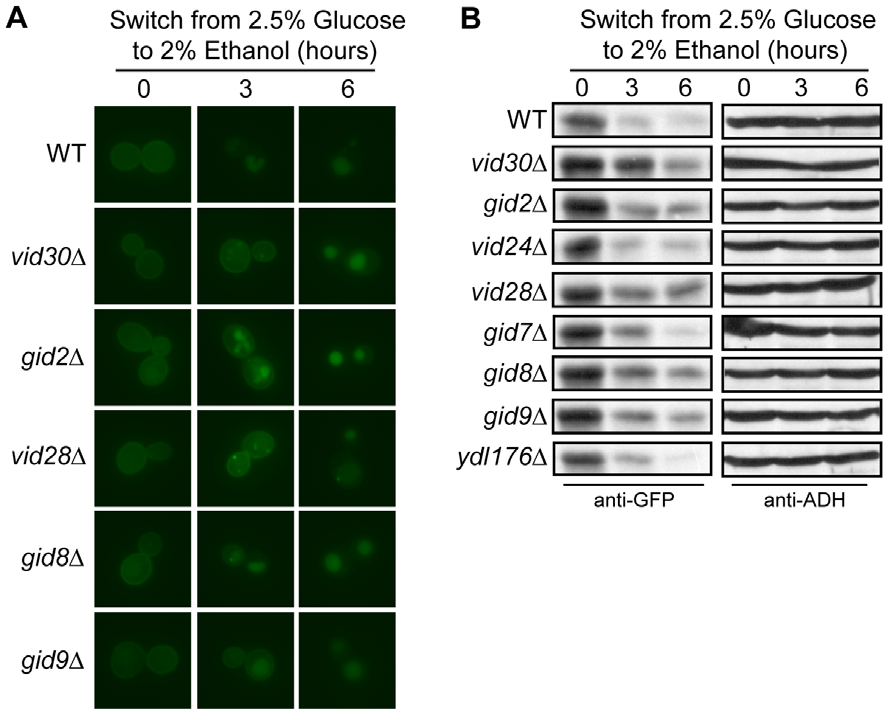
Components of the Vid30c are required for Hxt3 turnover. BY4742 (WT) and the indicated Vid30c mutants expressing *HXT3-GFP* were cultured in glucose media as described in the methods (time 0). Following a switch to ethanol media, samples were collected at the indicated times and analyzed by (A) fluorescence microscopy, and (B) Western analysis with anti-GFP antibodies. Identical blots were also probed with anti-ADH antibody as a loading control.

**Figure 2:**
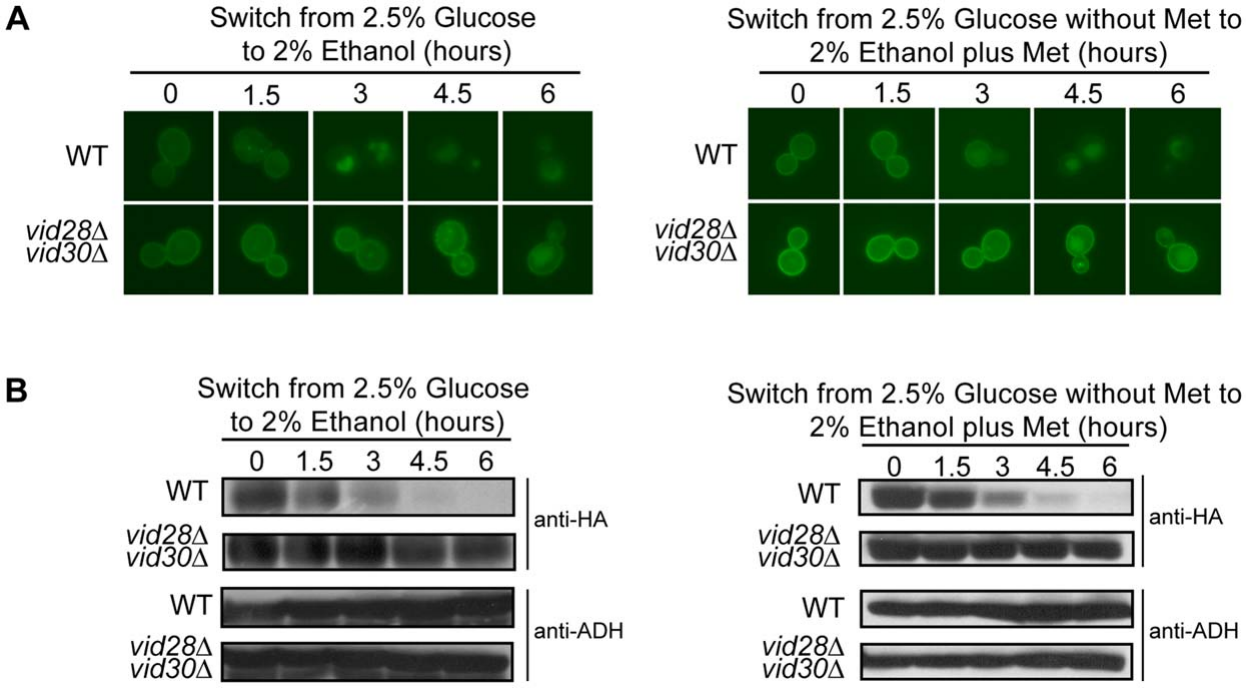
The combined deletion of *VID28* and *VID30* has an increased effect on Hxt3 turnover, which is maintained when the native promoter is exchanged for the *MET25* promoter. BY4742 (WT) and *vid28*Δ*vid30*Δ expressing *HXT3-GFP* from either the native promoter or the *MET25* promoter were cultured in glucose media (left) or glucose media minus methionine (right) as described in the methods (time 0). Following a switch to ethanol media (left) or ethanol media plus methionine (right), samples were collected at the indicated times and analyzed by (A) fluorescence microscopy, and (B) Western analysis with anti-GFP antibodies. Identical blots were also probed with anti-ADH antibody as a loading control.

## Materials and Methods

### Strains and Growth Conditions

All the yeast strains used in this study are isogenic to BY4742 and listed in Table S1. The chromosomally manipulated strains used in this study were created using the PCR-based integrative transformation procedure described previously [36]. The primers used contained 75 nt homologous to the native chromosomal locus up and downstream of the target integration site and 20 nt homologous to the specific template plasmid. The template plasmids were: pFA6a-GFP(S65T)-His3MX6 for the 3′ chromosomal fusion of the 3′ end of *HXT3* to *GFP* [36]; pYM-N35 (natMX-*MET25pro*) for the chromosomal fusion of the methionine-repressible *MET25* promoter (*MET25pro*) to the 5′ end of *HXT3* [37]; pYM-N4 (natMX-*CUP1pro*-*GFP*) for the chromosomal fusion of the copper-inducible *CUP1pro*-*GFP* cassette to the 5′ end of *HXT7* [37]; pFA6a-hphMX6 for the replacement of *VID30* with *hphMX6* [38]; and pCW1 (natMX-*PGK1pro*) for the replacement of the native *VID28* promoter with the constitutively active *PGK1* promoter. Following transformation, the correct integration events were verified by PCR. It is important to note that *HXT7* is a duplicated gene in the yeast genome with *HXT6* being its counterpart. The PCR confirmation of *HXT7* tagged strains therefore involved the use of an upstream primer in the upstream region of *HXT7* that is unique to *HXT7*. BY*tor1-1* was generated as previously described [39]. The proper point mutation was confirmed by sequencing.

**Figure 3:**
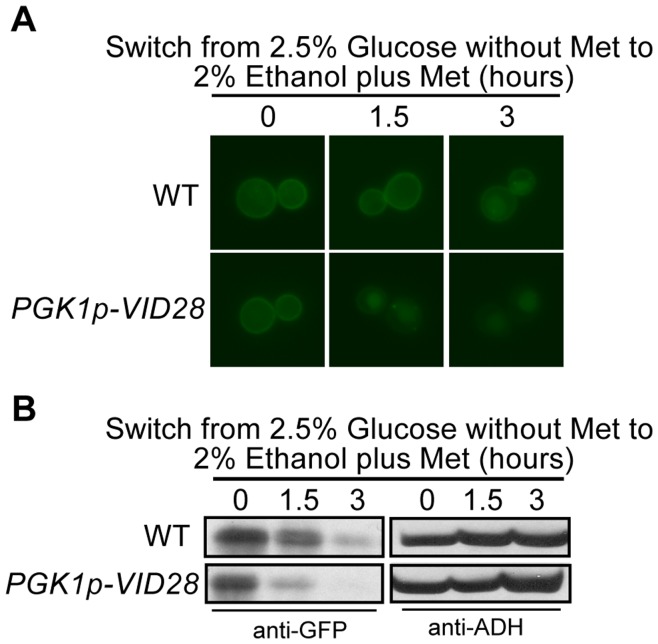
Overexpression of *VID28* accelerates Hxt3 turnover. BY4742 (WT) and *PGK1pro-VID28* expressing *MET25pro-HXT3-GFP* were cultured in glucose media minus methionine as described in the methods (time 0). Following washing and a switch to ethanol media plus methionine, samples were collected at the indicated times and analyzed by (A) fluorescence microscopy, and (B) Western analysis with anti-GFP antibodies. Identical blots were also probed with anti-ADH antibody as a loading control.

YCp50, YCp50-*RAS2* and YCp50-*RAS2^VAL19^* [40] were transformed into the indicated strains to determine the effects of the constitutively active *RAS^VAL19^* allele on Hxt turnover. Yeast strains used to monitor Hxt3-GFP localization and degradation were pre-cultured in synthetic complete media [0.17% Yeast Nitrogen Base (YNB) without amino acids and ammonium sulfate, 2% glucose, 0.5% ammonium sulfate and the CSM amino acid pool (minus uracil 0.77 g/L, or minus leucine 0.69 g/L, or minus methionine 0.75 g/L, MP Biomedicals)] to generate biomass. Cells were washed and transferred to synthetic media (0.17% YNB without amino acids and ammonium sulfate) containing 2.5% glucose and 0.5% ammonium sulfate. Amino acids were added to complement auxotrophic requirements. Following a three hour incubation to stimulate *HXT3* expression, cells were imaged or harvested for protein extraction (time zero). The remaining culture was harvested, washed and transferred to synthetic media (0.17% YNB without amino acids and ammonium sulfate) containing 2% ethanol and 0.5% ammonium sulfate. The 2% ethanol media was used to provide conditions in which glucose repression was alleviated; we will here after refer to its effect as “glucose starvation.” Amino acids were added to complement auxotrophic requirements and/or suppress expression from the *MET25* promoter. Samples were subsequently collected at the indicated times and used for fluorescence microscopy or protein extraction.

**Figure 4:**
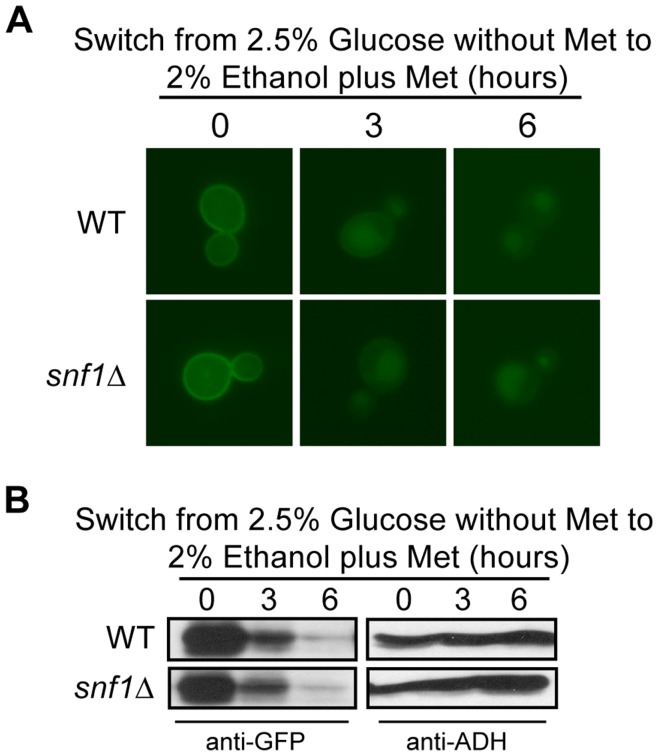
The function of Snf1 is not required for Hxt3 turnover. BY4742 (WT) and *snf1*Δ expressing *MET25pro-HXT3-GFP* were cultured in glucose media minus methionine as described in the methods (time 0). Following a switch to ethanol media plus methionine, samples were collected at the indicated times and analyzed by (A) fluorescence microscopy, and (B) Western analysis with anti-GFP antibodies. Identical blots were also probed with anti-ADH antibody as a loading control.

Yeast strains used to monitor GFP-Hxt7 localization and degradation were cultured as described in Snowdon *et al*. (2008) with the following exceptions: (1) the four hour pre-shift incubation was performed in raffinose with ammonium media containing 100 µM CuSO_4_ to stimulate *GFP-HXT7* expression from the *CUP1* promoter; (2) Cell samples were collected (time zero), and the remaining cells were washed twice with sterile water and resuspended in fresh raffinose with ammonium media devoid of CuSO_4_ followed by rapamycin treatment.

**Figure 5:**
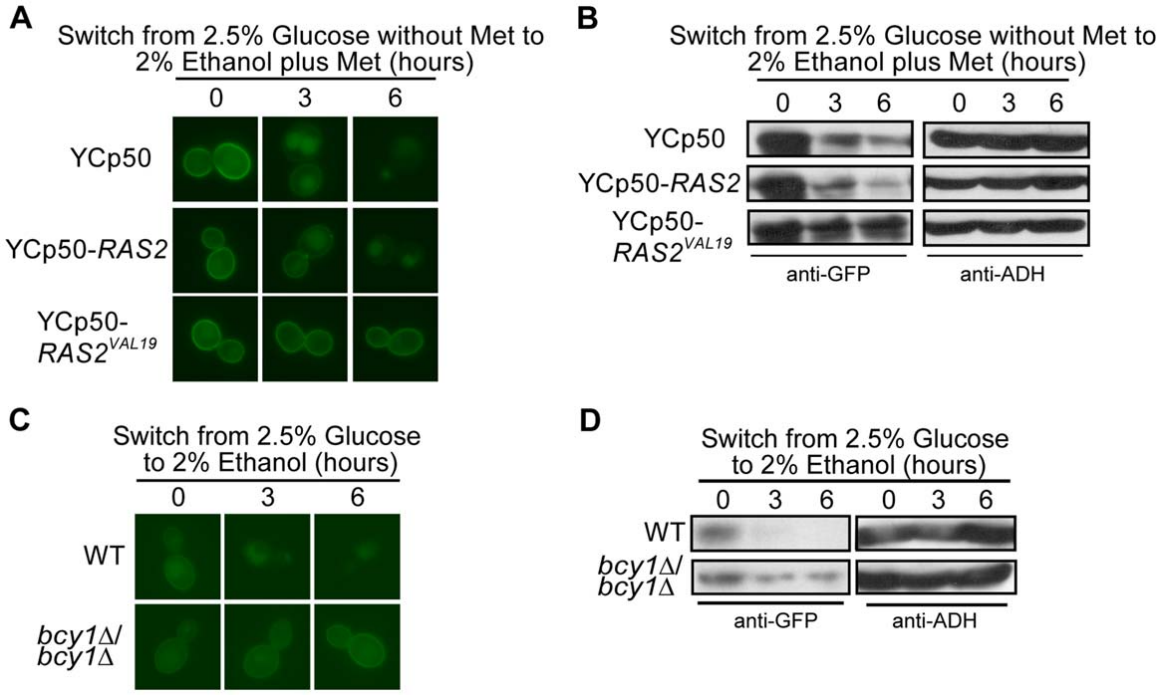
The turnover of Hxt3 requires inactivation of the Ras2/cAMP/PKA pathway. BY4742 expressing *MET25p-HXT3-GFP* was transformed with YCp50, YCp50-*RAS2* and YCp50-*RAS2^VAL19^*. Cells were cultured in glucose media minus methionine as described in the methods (time 0). Following a switch to ethanol media plus methionine, samples were collected at the indicated times and analyzed by (A) fluorescence microscopy, and (B) Western analysis with anti-GFP antibodies. Identical blots were also probed with anti-ADH antibody as a loading control. BY4743 (WT) and *bcy1*Δ/*bcy1*Δ strains expressing *HXT3-GFP* were cultured in glucose media as described in the methods (time 0). Following a switch to ethanol media, samples were collected at the indicated times and analyzed by (C) fluorescence microscopy, and (D) Western analysis with anti-GFP antibodies. Identical blots were also probed with anti-ADH antibody as a loading control.

### Fluorescence Microscopy

The monitoring of the subcellular localizations of the Hxt-GFP fusion proteins were performed by preparing slides directly from the indicated cell cultures followed by immediate analysis using the 100× objective lens of a Nikon Eclipse E600 microscope. Images were recorded using a Coolsnapfx monochrome CCD digital camera (Roper Scientific) and processed using Metamorph (Universal Imaging, Version 5.0).

**Figure 6:**
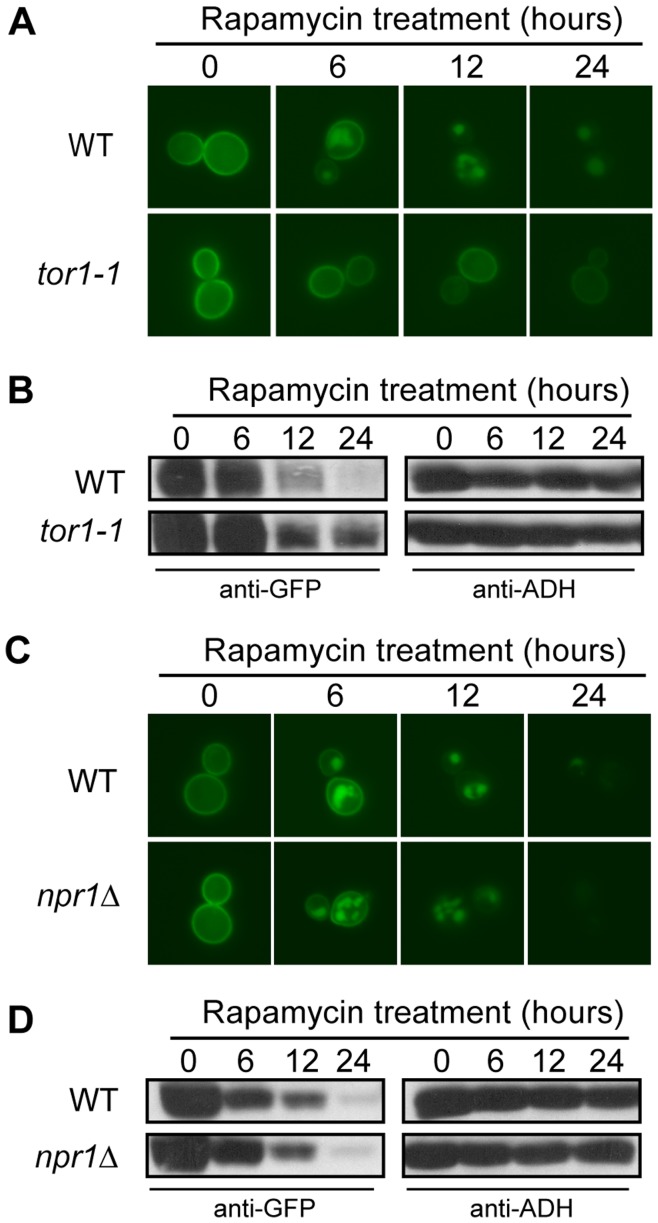
The rapamycin-induced turnover of Hxt7 signals through Tor1, but is Npr1-independent. (A) BY4742 and *tor1-1* expressing *CUP1pro-GFP-HXT7* were cultured in raffinose media plus CuSO_4_ as described in the methods (time 0). After harvesting and washing, the cells were resuspended in raffinose media devoid of CuSO_4_ and treated with rapamycin. Samples were collected at the indicated times and analyzed by (A) fluorescence microscopy and (B) Western analysis with anti-GFP antibodies. Identical blots were also probed with anti-ADH antibody as a loading control. BY4742 and *npr1*Δ strains expressing *CUP1pro-GFP-HXT7* were cultured and treated as outlined above. Samples were collected at the indicated times and analyzed by (C) fluorescence microscopy, and (D) Western analysis with anti-GFP antibodies. Identical blots were also probed with anti-ADH antibody as a loading control.

### Protein Extraction and Western Blotting

Harvested cells were resuspended in lysis buffer (1% NP40, 0.25% deoxycholate, 150 mM NaCl, 50 mM Tris pH 7.5, 1 mM EDTA, 1 mM PMSF; plus protease inhibitor cocktail tablets, Roche), added to 0.3 g glass beads and vortexed for two minutes. Lysates were centrifuged at 5,000 rpm for 3 minutes to remove cell debris. Supernatants were collected and the protein concentrations determined using the DC Protein Assay (Biorad) according to the manufacturer’s recommendations. Equal amounts of protein were separated by SDS-PAGE and transferred to nitrocellulose membranes. Mouse anti-GFP (Roche) and rabbit anti-Aldehyde Dehydrogenase (ADH) (200–4144, Rockland) were used as primary antibodies. ADH was used as the internal control to confirm equal amounts of protein in each lane as previously described [25], [41]. Horse radish peroxidase conjugates of donkey anti-mouse and donkey anti-rabbit immunoglobulin G (GE) were used as secondary antibodies. The ECL Detection kit (GE) was used to detect the secondary antibody according to the manufacturer’s recommendations. Membranes were exposed to autoradiography film for visualization.

**Figure 7:**
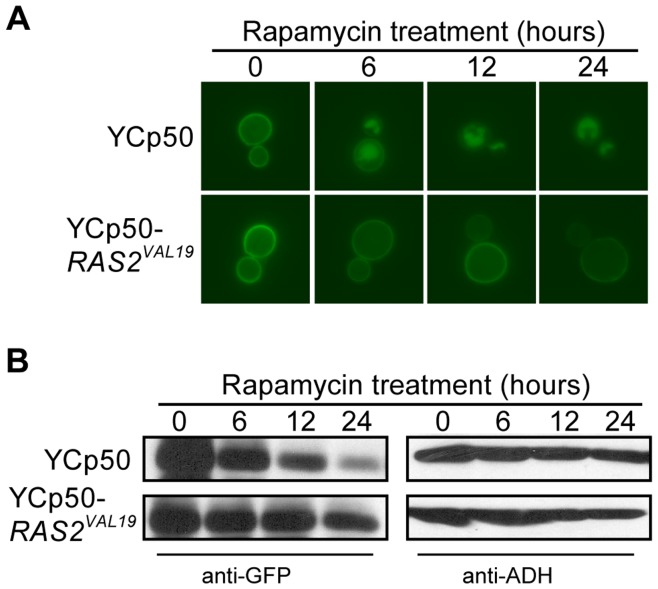
The rapamycin-induced turnover of Hxt7 requires inactivation of Ras2. BY4742 expressing *CUP1pro-HXT7-GFP* was transformed with YCp50 and YCp50-*RAS2^VAL19^*. Cells were cultured in raffinose media plus CuSO_4_ as described in the methods (time 0). After harvesting and washing, the cells were resuspended in raffinose media and treated with rapamycin. Samples were collected at the indicated times and analyzed by (A) fluorescence microscopy, and (B) Western analysis with anti-GFP antibodies. Identical blots were also probed with anti-ADH antibody as a loading control.

**Figure 8:**
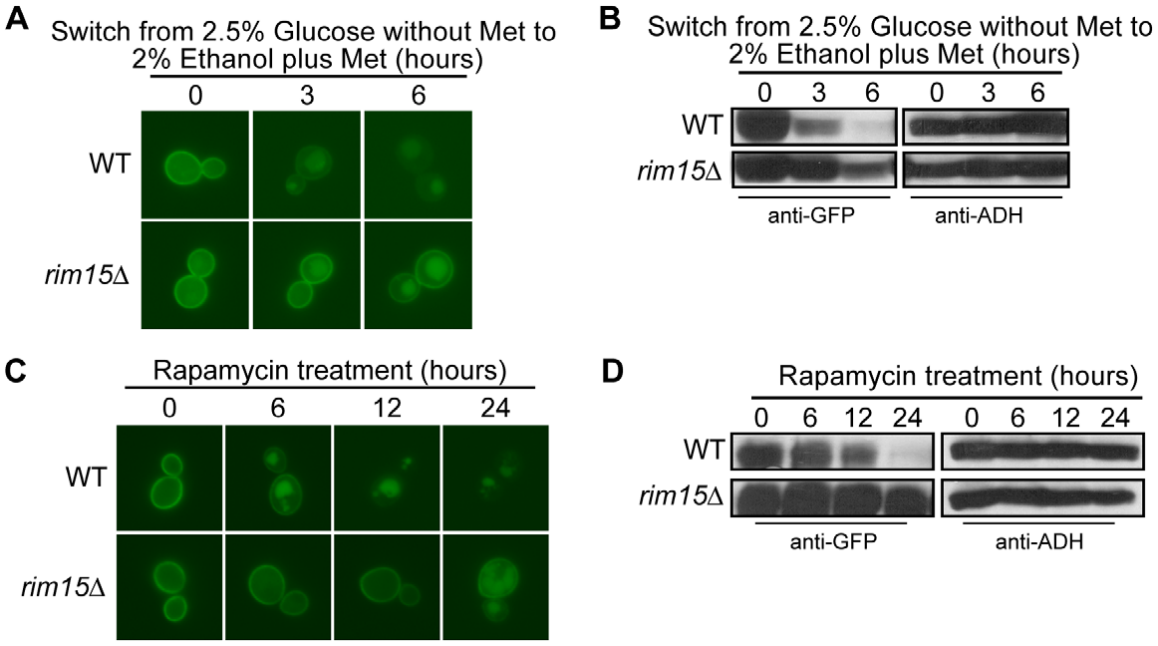
The turnover of both Hxt3 and Hxt7 is dependent on Rim15. BY4742 (WT) and *rim15*Δ expressing *MET25pro-HXT3-GFP* were cultured in glucose media minus methionine as described in the methods (time 0). Following a switch to ethanol media plus methionine, samples were collected at the indicated times and analyzed by (A) fluorescence microscopy, and (B) Western analysis with anti-GFP antibodies. Identical blots were also probed with anti-ADH antibody as a loading control. BY4742 and *rim15*Δ expressing *CUP1pro-GFP-HXT7* were cultured in raffinose media plus CuSO_4_ as described in the methods (time 0). After harvesting and washing, the cells were resuspended in raffinose media devoid of CuSO_4_ and treated with rapamycin. Samples were collected at the indicated times and analyzed by (C) fluorescence microscopy, and (B) Western analysis with anti-GFP antibodies. Identical blots were also probed with anti-ADH antibody as a loading control.

## Results

### Vid30c is Needed for the Ethanol-induced Regulation of Hxt3

During the adaptation to the absence of glucose in the environment, yeast represses the transcription of *HXT3* [26] and targets Hxt3 for endocytosis and ultimately degradation in the vacuole [27]. Since the Vid30c is needed for the nitrogen starvation-induced internalization and degradation of Hxt7 [25] we reasoned that this complex might also participate in the turnover of Hxt3 during glucose starvation, i.e. when glucose is replaced with ethanol as the sole carbon source. Wild type and individual Vid30c component mutant cells carrying *HXT3-GFP* were grown in glucose media to activate the expression of *HXT3*, and Hxt3-GFP was monitored upon a switch to ethanol as the sole carbon source. In glucose (time zero) *HXT3* transcription was activated and Hxt3-GFP was visible on the plasma membrane in all the strains tested (Figure S1A and Figure 1A). In the wild type strain Hxt3-GFP was internalized with Hxt3-GFP faintly visible on the plasma membrane 3 hours after the switch to ethanol media and almost completely degraded after 6 hours. By contrast, when monitoring the mutant strains we observed delayed internalization and degradation of Hxt3-GFP in the *vid30*Δ, *gid2*Δ, *vid28*Δ, *gid8*Δ and *gid9*Δ mutants as Hxt3-GFP displayed a delayed internalization from the plasma membrane (Figure 1A) or was clearly more abundant (Figure 1B) than in the wild type strain following the shift to ethanol media. The turnover of Hxt3-GFP in the *vid24*Δ, *gid7*Δ and *ydl176*Δ mutants was similar to that in the wild type (Figure 1B). Furthermore, our previous findings suggested that Vid28 and Vid30, the proposed core components of the Vid30c [28], had partially overlapping functions since the *vid28*Δ*vid30*Δ double mutant delayed Hxt7 turnover more efficiently than either of the respective single mutants [25]. Consistently, the glucose starvation-induced internalization and degradation of Hxt3-GFP in the *vid28*Δ*vid30*Δ strain was severely delayed in comparison to the respective single mutants (Figures 1A and 2A). These results were confirmed by western analyses (Figures 1B and 2B). In combination these data implicate the Vid30c in the turnover of two different Hxts, Hxt3 and Hxt7 [25], in response to different nutrient stimuli.

**Figure 9:**
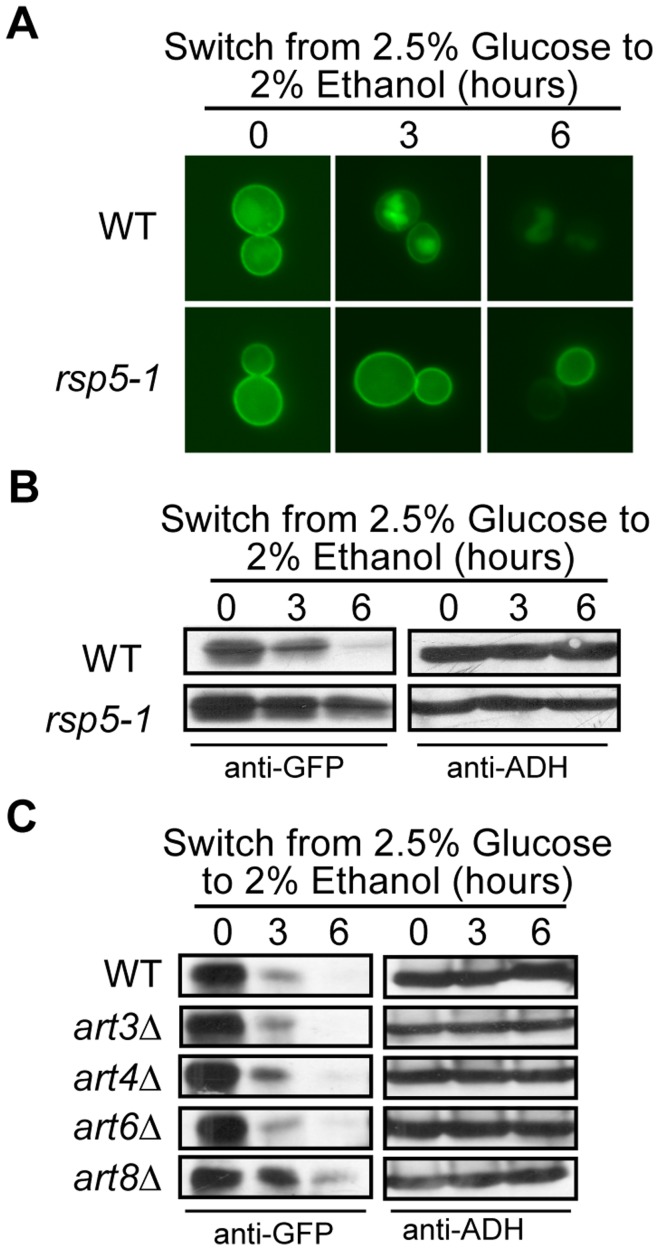
The turnover of Hxt3 is dependent on Rsp5 and Art8. (A and B) BY4742 (WT) and *rsp5-1*, and (C) BY4742 (WT), *art3*Δ, *art4*Δ, *art6*Δ and *art8*Δ expressing *HXT3-GFP* were cultured in glucose media as described in the methods (time 0). Following a switch to ethanol media, samples were collected at the indicated times and analyzed by (A) fluorescence microscopy, and (B and C) Western analysis with anti-GFP antibodies. Identical blots were also probed with anti-ADH antibody as a loading control.

The delayed protein turnover observed in the *vid*/*gid* mutants could be due to a lack of *HXT3-GFP* transcriptional repression after the shift from glucose to ethanol as the sole carbon source. When analyzing these strains for the transcriptional repression of *HXT3-GFP* we noticed slight, but reproducibly higher levels of *HXT3-GFP* mRNA in the *vid30*Δ, *gid2*Δ, *vid24*Δ, *vid28*Δ, *gid8*Δ and *vid28*Δ*vid30*Δ strains compared to the wild type after 3 hours in ethanol media (Figs. S1A and S1B). These findings suggest that the Vid30c may also have a minor impact on the efficient repression of *HXT3* transcription, or potentially mRNA stability, in the absence of glucose.

To separate the glucose starvation-mediated transcriptional repression of *HXT3* from Hxt3 turnover, we tested Hxt3 turnover with *HXT3-GFP* expression controlled by the methionine repressible *MET25* promoter [37] in the wild type and *vid28*Δ*vid30*Δ strains. Cells were grown to exponential phase in glucose media devoid of methionine to induce *HXT3-GFP* expression, washed to remove glucose, and transferred to ethanol media containing methionine to repress *HXT3-GFP* transcription. We confirmed the severely delayed glucose starvation-induced internalization and degradation of Hxt3 in the *vid28*Δ*vid30*Δ double mutant were similar regardless of *HXT3*-*GFP* being expressed from the native *HXT3* or *MET25* promoter (Figures 2A and 2B; right panels). The Vid30c is therefore specifically involved in the turnover of Hxt3, independent of transcriptional regulation.

The absence of *VID28* had the most severe delay of all the single *vid*/*gid* mutants on Hxt3 internalization and degradation (Figure 1). This prompted us to test the impact of the overexpression of *VID28* on Hxt3-GFP turnover. The native promoter of *VID28* was replaced with the constitutively active *PGK1* promoter in the *MET25pro-HXT3-GFP* strain. The overexpression of *VID28* showed a clear accelerated internalization and degradation of Hxt3-GFP following a shift from glucose to ethanol (Figures 3A and 3B). The localization of Hxt3-GFP to compartments of the endocytic pathway appears earlier and the Hxt3-GFP levels decrease quicker in the *PGK1pro-VID28* strain than in the wild type strain. Thus, the increased expression of *VID28* antagonizes the stability of Hxt3 in the plasma membrane. In combination these results confirm the participation of the Vid30c in the regulation of gene expression and protein turnover of Hxt3 as the cell adapts to ethanol as the sole carbon source.

### Active Ras/cAMP/PKA Prevents Ethanol-induced Hxt3 Turnover

Several signaling pathways enable yeast cells to respond to glucose availability. Snf1 is a central kinase known for its roles in the transcriptional activation of glucose-repressed genes, and the turnover of several target proteins during the adaptation to non-fermentable carbon sources in a glycolytic to gluconeogenic shift [42], [43]. The degradation of Hxt3 upon a switch from abundant glucose to ethanol media [27], led us to investigate the role of Snf1 in this event. Surprisingly, the wild type and *snf1*Δ mutant strains showed similar patterns of protein internalization and degradation (Figures 4A and 4B). These data indicate that Snf1 is not needed for the internalization and degradation of Hxt3-GFP in response to ethanol as a sole carbon source.

The Ras/cAMP/PKA signaling pathway plays a major role in controlling the response of yeast to glucose in the environment [44]. The activation of Ras1/2 occurs in response to abundant glucose in the environment and stimulates the adenylate cyclase Cyr1 to produce cAMP, which in turn activates PKA [18]. A constitutively active allele of *RAS2* (*RAS2^VAL19^*) renders PKA constitutively active [45]. We hypothesized that the inactivation of PKA is needed for the turnover of Hxt3-GFP and tested if constitutively active Ras2^Val19^ would impact the turnover of Hxt3-GFP in a shift to ethanol. Using the *MET25pro-HXT3-GFP* strain, our fluorescence microscopy and western analysis showed that the native *RAS2* allele supported the internalization of Hxt3-GFP at 3 hours and almost complete degradation in the vacuole 6 hours after an ethanol shift, while Ras2^Val19^ stabilized Hxt3 in the plasma membrane even after 6 hours in ethanol media with little, if any, internalization and degradation visible throughout the entire time course (Figures 5A and 5B). Active Ras2 therefore prevents the turnover of Hxt3.

Bcy1 binds and inactivates PKA in the absence of glucose [15]. Consequently, PKA is constitutively active in the absence of *BCY1* [46]. We analyzed the turnover of Hxt3 in the *bcy1*Δ/*bcy1*Δ mutant and, similar to the observations for the constitutively active Ras2^Val19^, no internalization or degradation of Hxt3 was observed in the *bcy1*Δ/*bcy1*Δ mutant (Figures 5C and 5D). Collectively these results demonstrate that the inactivation of Ras and PKA is integral to the signaling of Hxt3 turnover.

### Tor1 and Ras2, but not Npr1, Function in the Rapamycin-induced Turnover of Hxt7

The nitrogen starvation and rapamycin-induced turnover of amino acid permeases is known to be controlled by the Npr1 and Tor1 kinases in the TORC1 pathway [47], [48]. We used the rapamycin-insensitive *tor1-1* strain, carrying the *CUP1pro-GFP-HXT7* allele, to further analyze the previously reported rapamycin-induced degradation of Hxt7 [25]. The *CUP1pro-GFP-HXT7* allele replaces the native *HXT7* promoter with the copper inducible *CUP1* promoter to eliminate the impact of TOR signaling on *HXT7* transcription [25]. Our results indicate that the *tor1-1* allele impairs Hxt7 internalization and degradation (Figures 6A and 6B). We next tested if Hxt7 turnover was controlled by Npr1 and found the internalization and degradation profiles in response to rapamycin treatment to be similar in the wild type and *npr1*Δ mutant strains (Figures 6C and 6D). Collectively, these results suggest that the rapamycin-induced degradation of Hxt7 is TORC1-regulated, but in a manner independent of Npr1.

Several studies have shown close interactions between the TOR and Ras/cAMP/PKA pathways ranging from TOR and Ras/cAMP/PKA converging as separate pathways on the same molecular target [14], TOR functioning to control PKA as a downstream effector [49] to TOR and PKA having antagonistic effects in the cell [50]. Since Hxt3 turnover is controlled by Ras/cAMP/PKA (Figure 5) and TOR controls rapamycin-induced Hxt7 turnover in a mechanism independent of Npr1 (Figure 6), we tested the involvement of the Ras/cAMP/PKA pathway in rapamycin-induced Hxt7 turnover. While cells expressing native *RAS2* displayed normal rapamycin-induced turnover of GFP-Hxt7, the protein clearly remained in the plasma membrane with limited if any internalization following rapamycin treatment of cells expressing constitutively active *RAS2^VAL19^* (Fig. 7A). Higher levels of GFP-Hxt7 were also detected by western analysis in *RAS2^VAL19^* strains post rapamycin treatment (Figure 7B). These observations suggest that, like glucose starvation-induced Hxt3 turnover, the rapamycin-induced internalization and degradation of Hxt7 is dependent on the inactivation of Ras2.

### Rim15 Function is Required for the Turnover of Hxt3 and Hxt7

PKA and TOR control the activity of several downstream effectors in the presence of abundant glucose and nitrogen, respectively; a common downstream effector of both pathways is the Rim15 kinase [14], [51]. We investigated the potential role of Rim15 in the turnover of Hxt3 and Hxt7. While the levels of Hxt3-GFP and its localization to the plasma membrane were comparable in the wild type and *rim15*Δ mutant grown in glucose (time zero), the internalization of Hxt3-GFP in *rim15*Δ cells was markedly delayed when the cells were shifted to ethanol; the protein was still visible in the plasma membrane and abundantly present after 6 hours in ethanol (Figures 8A and 8B). Similarly, GFP-Hxt7 was expressed at similar levels and localized to the plasma membrane in the wild type and *rim15*Δ mutant strains grown in raffinose, but the *rim15*Δ mutant showed a clear delay in internalization and degradation when the cells were treated with rapamycin (Figures 8C and 8D). In combination these results demonstrate that active Rim15 is needed for glucose starvation and rapamycin-induced internalization and degradation of Hxt3 and Hxt7, respectively.

### Turnover of Hxt3 is Dependent on Rsp5 and Art8

The endocytosis of several amino acid permeases and hexose transporters is dependent on ubiquitylation by the E3 ubiquitin ligase Rsp5 [24], [33], [52], [53]. Members of the Art family of arrestin-like proteins recruit Rsp5 to nutrient transporters targeted for endocytosis [54], [55]. It is not known if Rsp5 or which of the Arts are needed for the internalization and subsequent degradation of Hxt3 and Hxt7. We analyzed the glucose starvation-induced internalization and degradation of Hxt3-GFP in the *rsp5-1^ts^* mutant. As this strain dies with prolonged exposure to ethanol at 37°C, we used 30°C as the non-permissive temperature. It was clear that Hxt3-GFP remained on the plasma membrane with no discernible internalization occurring even after 6 hours in ethanol (Figure 9A). The lack of protein degradation was also confirmed by western (Figure 9B). These results confirm Rsp5 as a major E3 ubiquitin ligase needed for the turnover of Hxt3. The glucose-induced turnover of Hxt7 is known to be dependent on Rsp5 [24], [56]. Similarly, we found that the rapamycin-induced endocytosis and subsequent degradation of Hxt7 is also dependent on Rsp5 (Figure S2A).

We screened four arrestin-like proteins, Art3, Art4, Art6, and Art8 for its potential involvement in Hxt3 and Hxt7 endocytosis and found that only the *art8*Δ mutant delayed Hxt3 turnover (Figure 9C and Figure S2B). The endocytosis of Hxt3-GFP was still observed in the absence of *ART8*, but to a much lesser extent than in the wild type strain, suggesting that other Rsp5 adaptor proteins, in addition to Art8, might be needed for the endocytosis of Hxt3. None of these *art* mutants impacted Hxt7 turnover (Figure S2C).

## Discussion

The Vid30c is needed for the adaptation of yeast to glucose replenishment following growth with gluconeogenic carbon sources (such as ethanol or acetate) as it functions as an E3 ubiquitin ligase responsible for the ubiquitylation and subsequent degradation of FBPase and Mdh2 when these enzymes are no longer needed [30], [32], [57]. Here we show components of the Vid30c also play an important role in the adaptation to gluconeogenic growth conditions following growth with glucose as the sole carbon source. *HXT3* is actively expressed and Hxt3 localizes to the plasma membrane when glucose is abundant, but *HXT3* transcription is repressed [26] and the protein is endocytosed and degraded when ethanol is the sole carbon source [27]. This latter adaptation depends on the Vid30c, more specifically the proposed core components Vid28, Vid30 and Gid8 [28] and the two RING finger proteins Gid2 and Gid9 [29], [30], as the transcriptional repression of *HXT3* and more noticeably the turnover of Hxt3 are delayed in the absence of these *VID*/*GID* genes (Figure S1; Figures 1 and 2). Components of the Vid30c are also needed for the nitrogen starvation-induced degradation of Hxt7 [25] and nitrogen-regulated gene expression [58]. Several components of the Vid30c therefore do not function solely in the adaptation of yeast to glucose replenishment, but are important for its adaptation to a range of nutrient conditions, indicating a more central role for this complex in the nutrient adaptation of eukaryotic cells.

Several signaling pathways enable the molecular response to the presence or absence of nutrient abundance in the environment. The Ras/cAMP/PKA pathway facilitates the cellular response and proliferation when glucose is abundant [59]–[61]. Similarly, the TORC1 pathway is active and supports cell cycle progression in rich nutrient, including nitrogen, conditions [4], [62], [63]. By contrast, the Snf1 pathway is activated when glucose is depleted and the cell experiences gluconeogenic growth conditions [42], [43], [61]. Through the manipulation of genes required for glucose and nitrogen signaling, we were able to delineate the role of each in the regulatory events required for the degradation of both Hxt3 and Hxt7. Since Hxt3 is endocytosed and degraded in gluconeogenic growth conditions, it was surprising to find that Snf1 did not have a role in this process. Likewise, Npr1 is a TORC1-controlled kinase known to be involved in the endocytosis and subsequent degradation of several amino acid permeases in a nitrogen-dependent manner [33], [52], [53]. We confirm that the previously reported rapamycin-induced degradation of Hxt7 [25] is dependent on Tor1, but surprisingly Npr1 is not involved in this process. Since the rapamycin-insensitive *tor1-1* allele prevented the endocytosis and degradation of Hxt7 in response to rapamycin treatment and the activation of the Ras/cAMP/PKA pathway is known to suppress Tor deficiencies [13], we hypothesized that active PKA would similarly prevent the turnover of Hxt3 and Hxt7. The predominant retention of Hxt3-GFP in the plasma membrane in ethanol when PKA is constitutively active in cells either expressing Ras2^Val19^ or lacking *BCY1* supports this hypothesis. Similarly, the rapamycin-induced internalization of Hxt7 is largely prevented in cells expressing Ras2^Val19^. Furthermore, in rich nutrient conditions in the absence of stress, both TORC1 and PKA prevent cell cycle arrest by inactivating Rim15, the kinase that promotes entry into G_0_ [14]. Inactivation of either TORC1 with rapamycin or PKA by growth with gluconeogenic carbon sources, results in the activation of Rim15 [14], [51], [64]. Our data show that active Rim15 is needed for the turnover of Hxt3 in ethanol and Hxt7 in response to rapamycin treatment. Collectively, our observations conclude that the Ras/cAMP/PKA pathway needs to be inactivated to enable the turnover of Hxt3 and Hxt7 in response to ethanol and rapamycin treatment. To our knowledge this is the first report of Rim15 participating in nutrient-regulated protein turnover. It is clear that Rim15 has a partial role facilitating Hxt3 and Hxt7 turnover. Other kinases could therefore potentially participate in signaling these turnover events. To this end, the Hal4 and Hal5 kinases are related to Npr1 and have recently been shown to participate in nutrient transporter turnover [65], [66]. However, unlike Npr1, the Hal4 and Hal5 kinases do not seem to function in response to nitrogen starvation or rapamycin treatment [65]. Nonetheless, it would be of interest to identify which kinases, in addition to Rim15, participate in the condition-specific turnover of Hxt3 and Hxt7.

Rsp5 is an essential E3 ubiquitin ligase responsible for the ubiquitylation and subsequent endocytosis of nutrient transporters [24], [33], [34], [52]. An assortment of arrestin-like proteins can function as adaptors for recruiting Rsp5 to nutrient transporters targeted for degradation [54], [55]. Here we show that Hxt3 endocytosis in response to glucose starvation is dependent on Rsp5 and Art8, confirming Rsp5 and the arrestin-like adaptors as major players needed for the endocytosis of Hxt3. The glucose-induced degradation of Hxt7 is known to be dependent on Rsp5 [24], [56], and here we confirm that the rapamycin-induced degradation is as well. Interestingly, Art8 was not needed for the endocytosis of Hxt7, suggesting that specificity exists between the Art adaptors and its specific hexose transporter target proteins. Importantly, very little, if any, turnover of Hxt3 occur in the *rsp5-1* ^ts^ mutant, confirming Rsp5 as an essential E3 ubiquitin ligase in Hxt3 turnover. We could not use the *rsp5-1^ts^* allele in the turnover of Hxt7 as the strain could not tolerate rapamycin treatment during the turnover experiment (data not shown). We therefore used the less dominant *rsp5-3* ^ts^ allele to study Hxt7 turnover. Hxt7 was clearly stabilized in the plasma membrane, but some internalization was observed. These observations implicate two E3 ubiquitin ligases, Rsp5 and the Vid30c, in the nutrient-mediated endocytosis and degradation of Hxt3 and Hxt7. Nutrient transporters have been shown to be direct targets for Rsp5-mediated ubiquitylation, suggesting a more indirect function for the Vid30c where it does not directly ubiquitylate the target Hxt. It is therefore of great importance to identify the precise role the Vid30c in Hxt turnover. Investigation into the signaling surrounding Hxt turnover has implicated the Ras/cAMP/PKA pathway and Rim15 in both unique turnover events. The strikingly similar retention of Hxt3 and Hxt7 in the plasma membranes of the *vid28*Δ*vid30*Δ double mutant and cells expressing Ras2^Val19^ could serve as the foundation to investigate a potential functional link between the Vid30c and the Ras/cAMP/PKA.

## Supporting Information

Figure S1 **Deletion of components of the Vid30c causes a slight increase in** ***HXT3*** **transcription.** BY4742 (WT), *vid30*Δ, *gid2*Δ, *vid28*Δ, *gid8*Δ, *gid9Δ* (A) and *vid28*Δ*vid30*Δ (B) expressing *HXT3-GFP* were cultured in glucose media as described in the methods (time 0). Following a switch to ethanol media, samples were collected at the indicated times and analyzed by northern blot analysis. Membranes were probed for *GFP* and *ACT1* as a loading control.
(TIF)

Figure S2 **The turnover of Hxt7 is dependent on Rsp5. BY4742 (WT) and** ***rsp5-3*** **(A) and BY4742 (WT),** ***art3*** **Δ,** ***art4*** **Δ,** ***art6*** **Δ and** ***art8*** **Δ (B) expressing** ***HXT7-GFP*** **were cultured in raffinose media as described in the methods, time 0.** After treated with rapamycin, samples were collected at the indicated times and analyzed by fluorescence microscopy (Top) and Western analysis with anti-GFP antibodies (Bottom). Identical blots were also probed with anti-ADH antibody as a loading control. (C) BY4742 (WT), *art3*Δ, *art4*Δ, *art6*Δ and *art8*Δ expressing *HXT3-GFP* were cultured in glucose media as described in the methods, time 0. Following a switch to ethanol media, samples were collected at the indicated times and analyzed by fluorescence microscopy.
(TIF)

Table S1 **Yeast strains used in this study.**
(DOCX)
