# Supplementary figures and images for: Regulation of Hxt3 and Hxt7 Turnover Converges on the Vid30 Complex and Requires Inactivation of the Ras/cAMP/PKA Pathway in Saccharomyces cerevisiae
Source: PLoS One. 2012 Dec 5;7(12):e50458. doi: 10.1371/journal.pone.0050458 (PMC3515616; doi:10.1371/journal.pone.0050458)

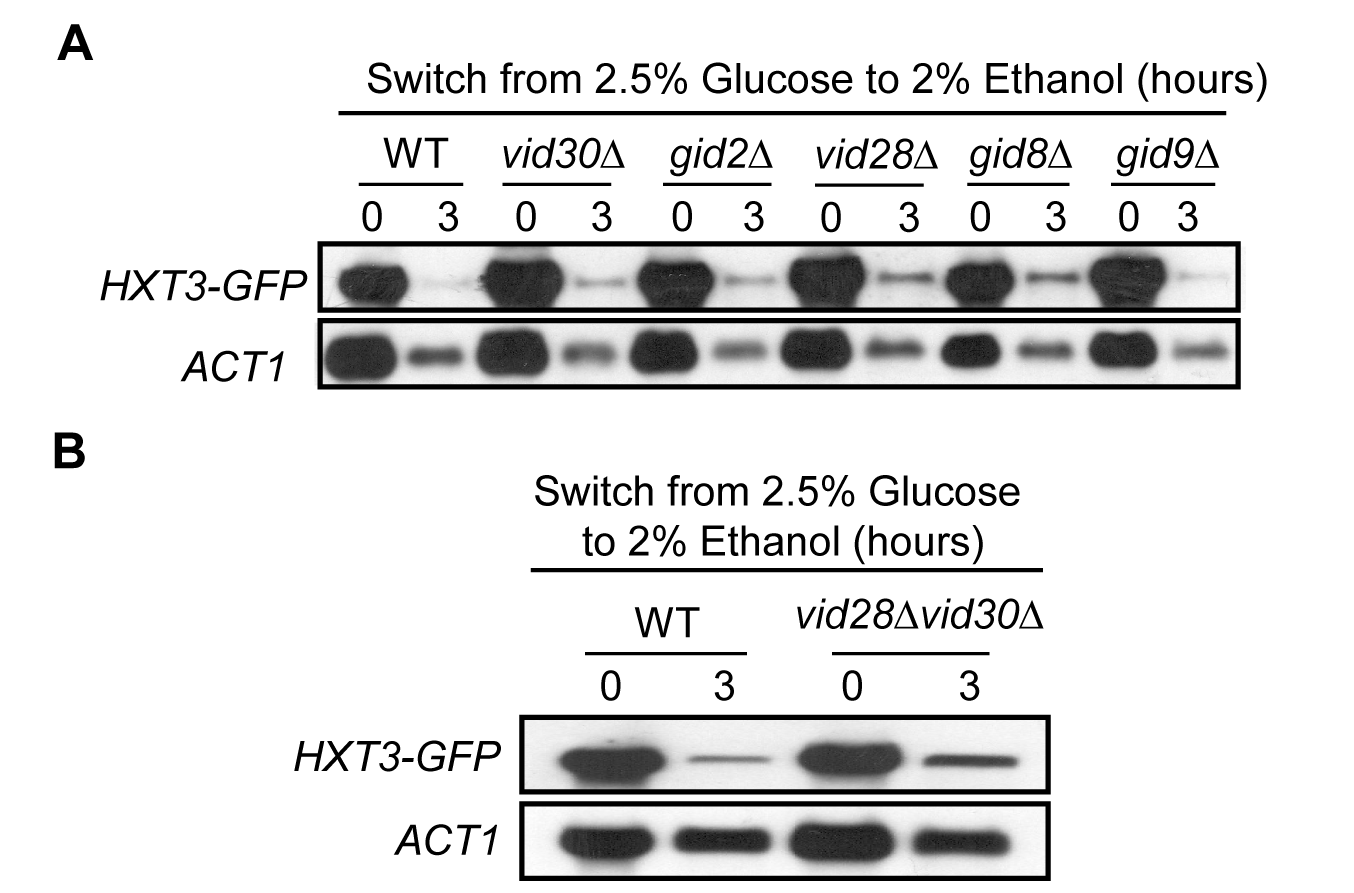

Supplement: Figure S1 — Deletion of components of the Vid30c causes a slight increase in HXT3 transcription. BY4742 (WT), vid30Δ, gid2Δ, vid28Δ, gid8Δ, gid9Δ (A) and vid28Δvid30Δ (B) expressing HXT3-GFP were cultured in glucose media as described in the methods (time 0). Following a switch to ethanol media, samples were collected at the indicated times and analyzed by northern blot analysis. Membranes were probed for GFP and ACT1 as a loading control. (TIF) [file pone.0050458.s001.tif]

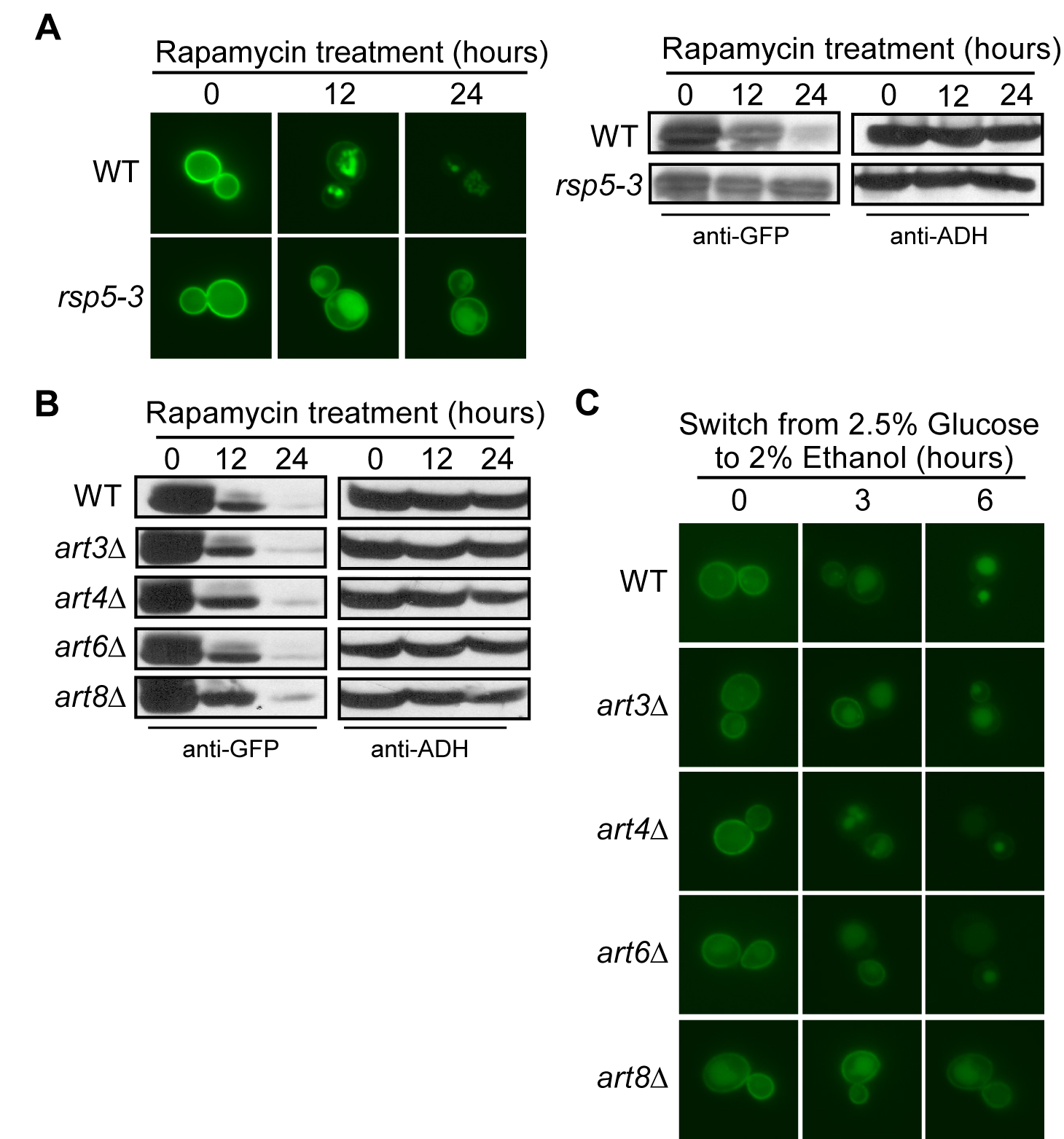

Supplement: Figure S2 — The turnover of Hxt7 is dependent on Rsp5. BY4742 (WT) and rsp5-3 (A) and BY4742 (WT), art3 Δ, art4 Δ, art6 Δ and art8 Δ (B) expressing HXT7-GFP were cultured in raffinose media as described in the methods, time 0. After treated with rapamycin, samples were collected at the indicated times and analyzed by fluorescence microscopy (Top) and Western analysis with anti-GFP antibodies (Bottom). Identical blots were also probed with anti-ADH antibody as a loading control. (C) BY4742 (WT), art3Δ, art4Δ, art6Δ and art8Δ expressing HXT3-GFP were cultured in glucose media as described in the methods, time 0. Following a switch to ethanol media, samples were collected at the indicated times and analyzed by fluorescence microscopy. (TIF) [file pone.0050458.s002.tif]
