# Supplementary material for: Regulation of Hxt3 and Hxt7 Turnover Converges on the Vid30 Complex and Requires Inactivation of the Ras/cAMP/PKA Pathway in Saccharomyces cerevisiae
Source: PLoS One. 2012 Dec 5;7(12):e50458. doi: 10.1371/journal.pone.0050458 (PMC3515616; doi:10.1371/journal.pone.0050458)
Supplement: Table S1 — Yeast strains used in this study. (DOCX) [file pone.0050458.s003.docx]

| Yeast strain | Genotype |
| --- | --- |
| BY4742 *HXT3-GFP*^b^ | *MATα* *his3∆1* *leu2∆0* *ura3∆0* *lys2∆0*^a^ *HXT3-GFP::His3MX6* |
| *vid30∆ HXT3-GFP*^b^ | BY4742 *vid30::KanMX4*^a^ *HXT3-GFP::His3MX6* |
| *gid2∆ HXT3-GFP*^b^ | BY4742 *gid2::KanMX4*^a^ *HXT3-GFP::His3MX6* |
| *vid24∆ HXT3-GFP*^b^ | BY4742 *vid24::KanMX4*^a^ *HXT3-GFP::His3MX6* |
| *vid28∆ HXT3-GFP*^b^ | BY4742 *vid28::KanMX4*^a^ *HXT3-GFP::His3MX6* |
| *gid7∆ HXT3-GFP*^b^ | BY4742 *gid7::KanMX4*^a^ *HXT3-GFP::His3MX6* |
| *gid8∆ HXT3-GFP*^b^ | BY4742 *gid8::KanMX4*^a^ *HXT3-GFP::His3MX6* |
| *gid9∆ HXT3-GFP*^b^ | BY4742 *gid9::KanMX4*^a^ *HXT3-GFP::His3MX6* |
| *ydl176w∆ HXT3-GFP*^b^ | BY4742 *ydl176w::KanMX4*^a^ *HXT3-GFP::His3MX6* |
| *vid28∆vid30∆ HXT3-GFP*^b^ | BY4742 *vid28::KanMX vid30::hphMX HXT3-GFP::His3MX6* |
| *snf1∆ HXT3-GFP*^b^ | BY4742 *snf1::KanMX4 natNT2::MET25pro-HXT3-GFP::His3MX6* |
| *rim15∆ HXT3-GFP*^b^ | BY4742 *rim15::KanMX4*^a^ *HXT3-GFP::His3MX6* |
| BY4743 *HXT3-GFP*^b^ | BY4743^a^ *HXT3-GFP::His3MX6* |
| *bcy1∆/bcy1∆* *HXT3-GFP*^b^ | BY4743 *bcy1::KanMX4/bcy1::KanMX4*^a^ *HXT3/HXT3-GFP::His3MX6* |
| *rsp5-1 HXT3-GFP*^b^ | BY4742 *rsp5-1 HXT3-GFP::His3MX6* |
| *art3∆ HXT3-GFP*^b^ | BY4742 *art3::KanMX4*^a^ *HXT3-GFP::His3MX6* |
| *art4∆ HXT3-GFP*^b^ | BY4742 *art4::KanMX4*^a^ *HXT3-GFP::His3MX6* |
| *art6∆ HXT3-GFP*^b^ | BY4742 *art6::KanMX4*^a^ *HXT3-GFP::His3MX6* |
| *art8∆ HXT3-GFP*^b^ | BY4742 *art8::KanMX4*^a^ *HXT3-GFP::His3MX6* |
| *MET25pro-HXT3-GFP*^b^ | BY4742^a^ *natNT2::MET25pro-HXT3-GFP::HIS3MX6* |
| *vid28∆vid30∆ MET25pro-HXT3-GFP*^b^ | BY4742 *vid28::KanMX*^a^ *vid30*::*hphMX natNT2::MET25pro-HXT3-GFP::HIS3MX6* |
| *rim15∆ MET25pro-HXT3-GFP*^b^ | BY4742 *rim15::KanMX4*^a^ *natNT2:MET25pro-HXT3-GFP::HIS3MX6* |
| *PGK1pro-VID28 MET25pro-HXT3-GFP*^b^ | BY4742^a^ *KanMX4::PGK1pro-VID28 natNT2::MET25pro-HXT3-GFP::HIS3MX6* |
| BY4742 *HXT7-GFP*^b^ | BY4742^a^ *HXT7-GFP::His3MX6* |
| *rsp5-3 HXT7-GFP*^b^ | BY4742 *rsp5-3 HXT7-GFP::His3MX6* |
| *art3∆ HXT7-GFP*^b^ | BY4742 *art3::KanMX4*^a^ *HXT7-GFP::His3MX6* |
| *art4∆ HXT7-GFP*^b^ | BY4742 *art4::KanMX4*^a^ *HXT7-GFP::His3MX6* |
| *art6∆ HXT7-GFP*^b^ | BY4742 *art6::KanMX4*^a^ *HXT7-GFP::His3MX6* |
| *art8∆ HXT7-GFP*^b^ | BY4742 *art8::KanMX4*^a^ *HXT7-GFP::His3MX6* |
| BY4742 *CUP1pro-GFP-HXT7*^b^ | BY4742^a^ *natNT2::CUP1pro-GFP-HXT7* |
| *tor1-1* *CUP1pro-GFP-HXT7*^b^ | BY4742 *tor1-1*^b^ *natNT2::CUP1pro-GFP-HXT7* |
| *npr1∆* *CUP1pro-GFP-HXT7*^b^ | BY4742 *npr1::KanMX4*^a^ *natNT2::CUP1pro-GFP-HXT7* |
| *rim15∆* *CUP1pro-GFP-HXT7*^b^ | BY4742 *rim15::KanMX4*^a^ *natNT2::CUP1pro-GFP-HXT7* |

^a^Open Biosystems

^b^This study
